# Preliminary Insights into the Seasonal Variation, Phylogenetic Diversity, and Biofilm-Forming Capacity of Cultivable *Vibrionaceae* in Coastal Biofilms of Qingdao, China

**DOI:** 10.3390/microorganisms14061259

**Published:** 2026-06-03

**Authors:** Leihaothabam Jeeny, Lingman Ran, Chukwuma Kenneth Chibuikem, Wen Hong, Yunqi Ding, Yan Wang, Yi Liu, Xiao-Hua Zhang, Xiaolei Wang

**Affiliations:** 1 Frontiers Science Center for Deep Ocean Multispheres and Earth System, College of Marine Life Sciences, Ocean University of China, Qingdao 266003, China; jeenyleihaothabam@gmail.com (L.J.);; 2 Laboratory for Marine Ecology and Environmental Science, Qingdao Marine Science and Technology Center, Qingdao 266237, China; 3 Key Laboratory of Evolution and Marine Biodiversity (Ministry of Education), Ocean University of China, Qingdao 266003, China

**Keywords:** *Vibrionaceae*, *Vibrio*, *Photobacterium*, biofilm formation, seasonality, coastal marine environment

## Abstract

Biofilm formation enables marine *Vibrionaceae* spp. to survive and adapt in dynamic coastal environments, yet their seasonal and substrate-related biofilm dynamics remain poorly understood. Our study investigated the seasonal distribution, biofilm-forming capacity, and phylogenetic diversity of cultivable *Vibrionaceae* isolated across four seasons from multiple marine substrates (sand, rocks, algae, and glass plate-associated biofilms) at Huiquan Beach, Qingdao. Biofilm formation was evaluated using a crystal violet microtiter plate assay, and taxonomic identification using 16S rRNA gene sequencing. A total of 176 *Vibrionaceae* isolates were identified, representing the *Splendidus* and *Harveyi* clades and a distinct *Photobacterium* lineage. Biofilm formation varied significantly with season and substrate type, with spring and summer isolates generally exhibiting stronger biofilm-forming capacity than the autumn and winter isolates. Stable substrates, such as algae and rocks, supported more consistent biofilm development than sand and glass plate-associated biofilms. Phylogenetic analysis suggested that biofilm-forming capacity was distributed across multiple clades, indicating strain-level variability. Notably, *Vibrio echinoideorum* was detected across all seasons and substrates, indicating ecological generalism. These findings provide insights into seasonal and substrate-associated patterns of cultivable *Vibrionaceae* in coastal biofilms. However, because environmental parameters were not directly measured, interpretations of environmental influences remain correlative.

## 1. Introduction

Marine ecosystems harbor highly diverse microbial communities that play essential roles in global biogeochemical cycles and ecosystem functioning [1] In coastal environments, microbial community structure is strongly influenced by physicochemical gradients and exhibits pronounced seasonal variability [2]. Marine bacteria adopt both planktonic and surface-associated lifestyles, forming biofilms embedded in a self-produced extracellular polymeric substance (EPS) matrix [3]. Biofilms enhance microbial survival by promoting surface attachment, protection from environmental stress, and cell–cell interactions [4]. In coastal ecosystems, biofilms develop on diverse substrates such as rocks, algae, and sediments, where they act as hotspots of microbial activity and biogeochemical processes [3,5].

Although members of the genus *Vibrio* are metabolically versatile and highly responsive to environmental fluctuations, marine microbial communities are generally dominated by other taxa, particularly *Alphaproteobacteria* (e.g., *Rhodobacteraceae*) and *Cyanobacteria* [6,7,8]. In this context, *Vibrio* represents an ecologically important and dynamic group rather than a universally dominant genus. This reflects their opportunistic lifestyle and rapid response to environmental changes, particularly in nutrient-rich coastal systems [6,7]. *Vibrios* exhibit rapid growth, high metabolic flexibility, and strong biofilm-forming ability, which enhance environmental survival, nutrient acquisition, and resistance to stressors [6]. These traits underpin their ecological success as efficient nutrient recyclers while simultaneously contributing to their emergence as opportunistic pathogens, positioning *Vibrio* species as model organisms for understanding microbial adaptation in dynamic coastal environments [6,9]. With more than 140 validated species, *Vibrio* spp. display remarkable genetic and ecological diversity across marine, estuarine, and aquaculture environments [10]. Phylogenetic analyses identified several major clades, including the *Cholerae*, *Vulnificus*, *Harveyi*, and *Splendidus* clades, each associated with distinct habitats and functional attributes [11,12]. In coastal biofilm communities, these clades frequently co-occur with environmentally abundant yet comparatively understudied lineages such as those related to *Vibrio splendidus* and *Vibrio lentus* [13], whose ecological roles and biofilm-forming capacities in temperate marine systems remain insufficiently characterized [5].

*Vibrio* species contribute to marine carbon and nitrogen cycling by degrading organic matter and rapidly utilizing transient nutrient inputs [6]. Their metabolic flexibility enables them to respond quickly to environmental variability, particularly in nutrient-rich coastal systems [14]. In addition to their ecological roles, some species are opportunistic pathogens, highlighting their ecological and functional duality [15]. Environmental factors, especially temperature and nutrient availability, strongly influence *Vibrio* population dynamics, with warmer conditions generally promoting increased abundance and extended seasonal occurrence [6,16,17].

Despite extensive research on planktonic *Vibrio* ecology, the seasonal dynamics of biofilm-associated populations in natural coastal environments remain poorly understood [5,18]. Most existing studies have focused on water-column communities, leaving biofilm-associated ecological patterns largely unresolved [19]. Moreover, the influence of substrate heterogeneity on *Vibrio* community assembly has received limited attention, as previous studies have predominantly focused on planktonic seawater isolates and have neglected algae, sediments, and rocks [5,20,21].

These substrates provide heterogeneous microhabitats differing in nutrient composition, surface properties, and physicochemical conditions that may strongly influence *Vibrio* colonization and persistence [6,22,23]. Consequently, it remains unclear whether biofilm-associated communities exhibit seasonal patterns similar to planktonic populations or are primarily structured by environmental and substrate-driven factors [24].

The Qingdao coast, located in the northwestern Yellow Sea, represents a typical temperate shallow continental coastal system situated between mainland China and the Korean Peninsula and influenced by seasonal monsoons, freshwater inputs, and intensive human activities [25]. The coexistence of diverse coastal substrates together with pronounced seasonal environmental gradients makes the Qingdao coast an ideal site for investigating seasonal dynamics and substrate-specific ecology of biofilm-forming vibrios. This study combined culture-based isolation, quantitative biofilm phenotyping, and phylogenetic analysis to characterize seasonal variation within cultivable bacterial communities isolated from Huiquan Beach (Qingdao), with a focus on *Vibrio* spp. The diversity and seasonal distribution of total bacteria and *Vibrio* species were examined across rocks, algae, sand, and glass plate-associated biofilm. Biofilm-forming capacity was quantified across seasons and incubation periods, and relationships between phylogenetic affiliation and biofilm phenotypes were evaluated to determine whether biofilm-forming capacity is phylogenetically conserved or environmentally driven. By examining representatives from multiple ecological lineages, including the *Splendidus*, *Harveyi*, *Mediterranean*, and *Orientalis* clades as well as *Photobacterium* spp., across contrasting substrates and seasonal cycles, this study provides a framework for evaluating environmental persistence, substrate adaptability, and biofilm-forming potential of vibrios, thereby advancing understanding of microbial community dynamics, nutrient cycling, and pathogen monitoring under ongoing climate change and anthropogenic pressures.

## 2. Materials and Methods

### 2.1. Study Description and Sampling Period

**Figure 1:**
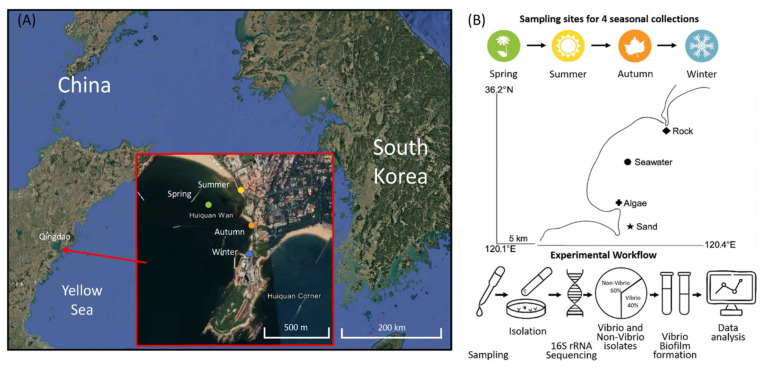
Sampling sites and experimental design. (**A**), Location of the sampling site at Huiquan Bay, Qingdao, China. The inset map shows the regional position of Qingdao along the Yellow Sea coast, while the main panel highlights Huiquan Bay with seasonal sampling locations (spring, summer, autumn, and winter). Base map modified from Google Earth Pro. (**B**), Overview of the experimental workflow used in this study.

The study area was Huiquan Bay (Huiquan Beach), located in Qingdao, China, on the western margin of the Yellow Sea (Figure 1A), at approximately 36.2° N and 120.1–120.4° E. The bay is a semi-enclosed coastal system influenced by tidal mixing, coastal currents, and anthropogenic inputs. The region is characterized by a temperate monsoon climate with marked seasonal fluctuations in temperature and hydrodynamic conditions. Huiquan Bay represents a suitable natural system for investigating seasonal dynamics of biofilm-associated *Vibrio* communities due to its pronounced environmental variability and diverse coastal substrates. Biofilm samples were collected seasonally from multiple substrates, including seawater, sand, rocks, and algae. Bacterial isolates were obtained using culture-based methods, identified by 16S rRNA gene sequencing, and assessed for biofilm formation using a crystal violet microplate assay after 24 h and 48 h of incubation. An overview of the experimental design is shown in Figure 1B.

Samples were collected seasonally in spring, summer, autumn, and winter from spring 2023 to January 2024 (winter 2024), with three replicates per substrate per season (n = 3). Sampling was conducted at fixed stations from four substrates: rock, algae, sand, and glass plate-associated biofilms. Rock samples were obtained by carefully scraping the surface layers using sterile scalpels and transferring the material into sterile containers. Algae samples were obtained by gently washing the algal surfaces with sterile seawater, followed by low-power sonication or gentle scraping to dislodge attached microorganisms without damaging the algae. Sand samples were collected from the surface layer, and microbes were separated from sediment particles through washing, vigorous shaking, and density gradient centrifugation.

Surface-associated microbial communities were collected using sterile glass plates, which were briefly immersed in seawater and then slowly withdrawn to capture the adhering microlayer. The collected material was transferred into sterile containers for further processing. These samples, therefore, represent biofilm-associated communities on an artificial substrate rather than planktonic seawater communities. This approach was used to standardize the sampling of surface-associated bacteria and to enable direct comparison with natural substrates such as rocks, algae, and sand.

Microbial suspensions from each substrate were plated on thiosulfate–citrate–bile salts–sucrose (TCBS) agar and marine 2216E agar (MA) (both from Qingdao Hopebio Technology Co., Ltd., Qingdao, China) to enable the isolation of vibrios and other heterotrophic marine bacteria. Plates were incubated at 28 °C for 24 h to 48 h. Colonies exhibiting characteristic *Vibrio*-like morphology on TCBS medium, as well as other bacterial colonies on MA, were selected for further purification. Colonies picked from both TCBS and MA plates were repeatedly streaked on fresh MA plates until pure cultures were obtained. MA was subsequently used for routine maintenance of all isolates and for downstream experiments, including biofilm formation assays.

### 2.2. Isolation and Purification of Cultivable Bacteria

The collected samples from each substrate were serially diluted using a sterile saline solution. For each sample, six 1.5 mL centrifuge tubes (Beijing Labgic Technology Co., Ltd., Beijing, China) were prepared, sterilized by autoclaving, and dried. Under aseptic conditions, 900 μL of sterile saline was added to each tube and appropriately labeled. To prepare the dilution series, 100 μL of each sample was added to the first EP tube and thoroughly mixed. Subsequently, 100 μL was transferred sequentially from the first to the sixth tube, resulting in serial dilutions spanning 10^−1^ to 10^−6^.

To enable the isolation of vibrios and other heterotrophic marine bacteria, 200 μL of each sample at 10^−2^, 10^−3^, and 10^−4^ was spread evenly on TCBS medium plates and incubated at 28 °C for 1–2 days. Similarly, 200 μL of samples from 10^−4^, 10^−5^, and 10^−6^ dilutions were plated on MA in triplicate and incubated at 28 °C for 2–3 days. These dilution ranges were selected based on preliminary experiments to obtain countable colonies (30–300 CFU per plate) for each substrate type. The lower dilutions (10^−2^–10^−4^) for TCBS account for its selective nature (which reduces colony counts), while the higher dilutions (10^−4^–10^−6^) for MA were necessary to avoid overgrowth on this non-selective medium. After incubation, morphologically distinct colonies were selected based on differences in shape, size, color, and surface characteristics. Each selected colony was streaked onto fresh MA plates using the three-zone streaking method until pure cultures were obtained. After 2–3 rounds of subculturing, pure isolates were preserved in marine broth supplemented with 20% (*v*/*v*) glycerol and stored at −80 °C for further analysis. MA was subsequently used for the routine maintenance of all isolates and downstream experiments.

### 2.3. Genomic DNA Extraction and Identification for Bacteria

Genomic DNA was extracted from freshly grown bacterial isolates using the boiling method. Briefly, a single colony was suspended in 200 µL of Tris–EDTA (TE) buffer in sterile 1.5 mL microcentrifuge tubes, vortexed, boiled at 100 °C for 10 min, immediately chilled at −20 °C for at least 30 min, and centrifuged at 12,000× *g* for 10 min at 4 °C. The resulting supernatant was used as the DNA template. The 16S rRNA gene was amplified by PCR using the universal primers 27F (5′-AGAGTTTGATCMTGGCTCAG-3′) and 1492R (5′-GGTTACCTTGTTACGACTT-3′), which were synthesized by Sangon Biotech (Shanghai) Co., Ltd. (Shanghai, China). PCR reactions (30 µL) contained 3.0 µL of buffer, 3.0 µL of dNTP mixture, 0.3 µL of each primer, 0.15 µL of DNA polymerase, 0.4 µL of template DNA, and nuclease-free water to bring the volume to 30 µL. Thermal cycling conditions were as follows: initial denaturation at 94 °C for 5 min, 30 cycles of denaturation at 94 °C for 1 min, annealing at 55 °C for 1 min, and extension at 72 °C for 1.5 min, followed by a final extension at 72 °C for 10 min. Reactions were then held at 25 °C.

PCR amplification was performed in a thermal cycler (model ALD1244, Bio-Rad Laboratories, Hercules, CA, USA). PCR products were verified by electrophoresis on 1% agarose gels, and amplicons showing a ~1500 bp band were selected for sequencing. Sanger sequencing was performed commercially (Beijing Liuhe BGI Technology Co., Ltd., Beijing, China). Chromatograms were checked and edited, and consensus sequences were generated. Sequences were compared to reference type strains using the EzBioCloud database (https://www.ezbiocloud.net, accessed in March 2023) for taxonomic identification. Taxonomic assignments followed widely accepted 16S rRNA gene similarity thresholds: ≥98.65% sequence similarity to a type strain was considered species-level identity, and isolates showing <98.65% similarity were considered as potentially species [26].

### 2.4. Biofilm Formation Assay

Biofilm formation was evaluated using a 96-well polystyrene microtiter plate assay (Nest Biotechnology Co., Ltd., Wuxi, China), following a modified protocol described by Coffey and Anderson (2014) [27]. Briefly, bacterial isolates were grown overnight in marine broth (MB) (Qingdao Hopebio Technology Co., Ltd., Qingdao, China) at 28 °C. Standardized aliquots of each culture were then inoculated into sterile 96-well polystyrene plates and incubated at 28 °C under static conditions for 24 h to allow biofilm development. After incubation, planktonic cells were gently removed, and each well was washed twice with sterile saline solution to eliminate non-adherent cells. The remaining attached biofilms were fixed and stained with 1% (*w*/*v*) crystal violet (Solarbio Life Sciences Co., Ltd., Beijing, China) for 15 min, then thoroughly washed with sterile saline to remove excess stain. Plates were air-dried at room temperature, and the bound crystal violet was solubilized using 95% ethanol. Biofilm formation was quantified by measuring absorbance at OD_570nm_ using a microplate reader (SpectraMax iD3s, Molecular Devices, San Jose, CA, USA). Each strain was tested in triplicate, and wells containing uninoculated medium served as negative controls. This assay enabled comparative evaluation of biofilm-forming capacity among *Vibrio* spp. and related isolates.

### 2.5. Phylogenetic Tree Construction

Sequences of 67 representative isolates were aligned using MAFFT (v7.526) with parameter ‘--maxiterate 1000 -localpair’ and then trimmed with Gblocks (v0.91b). A maximum-likelihood phylogenetic tree was constructed using IQ-TREE (v2.0.7) with parameter ‘-m MFP -alrt 1000 -bb 1000’. The best-fit nucleotide substitution model was the K3P+I+G4 model. The robustness of the inferred topology was assessed by SH-aLRT (Shimodaira–Hasegawa approximate likelihood ratio test) analysis with 1000 replicates. The final phylogenetic tree was visually enhanced using Tree Visualization Bot (TVBOT) for improved clarity and presentation [28]. Annotations in the tree indicate the season and substrate from which each isolate was obtained, as well as 16S rRNA gene sequence similarity (%) to the closest reference or type strain.

### 2.6. Data Analyses

All experimental data were compiled and organized using Microsoft Excel 2016. For the analysis of biofilm formation, ten isolates per season were randomly selected from those exhibiting detectable biofilm-forming capacity. This number was chosen to ensure a balanced comparison across seasons while maintaining statistical power and keeping the analysis practical. Seasonal differences in biofilm formation after 24 h and 48 h incubation were evaluated using the Kruskal–Wallis test, followed by Dunn’s multiple comparison test with Bonferroni correction, with statistical significance set at *p* < 0.05 [29,30] in SPSS (version 26). Statistical analyses were performed using SPSS (version 26). Substrate effects were analyzed using the same approach, but post-hoc comparisons were not conducted when the overall test was not significant. Taxonomic composition at the family and genus levels across seasons and substrates was analyzed descriptively. Data visualizations were generated using OriginPro 9.1 (2025), GraphPad 9.0 (GraphPad Software, Inc., San Diego, CA, USA), and Chiplot (https://chiplot.online/, accessed on 6 June 2025).

## 3. Results

### 3.1. Composition of Cultivable Bacterial Families

A total of 339 cultivable bacterial isolates were recovered from coastal biofilm samples collected across four seasons at Huiquan Bay. Based on nearly full-length 16S rRNA gene sequence analysis, these isolates were classified into four phyla, five classes, ten orders, and sixteen families. At the phylum level, the cultivable community was strongly dominated by *Pseudomonadota* (87.61%), followed by *Bacillota* (8.85%), *Bacteroidota* (3.24%), and *Actinomycetota* (0.29%) (Figure S1A). At the class level, *Gammaproteobacteria* accounted for the majority of isolates (81.42%), with smaller contributions from *Bacilli* (8.85%), *Alphaproteobacteria* (6.19%), *Flavobacteriia* (3.24%), and *Actinomycetes* (0.29%) (Figure S1B). Order-level analysis revealed *Vibrionales* as the dominant group (51.92%), followed by *Alteromonadales* (18.58%) and *Pseudomonadales* (8.85%) (Figure S1C). At the family level, *Vibrionaceae* was the most abundant family (52.23%), followed by *Alteromonadaceae* (9.79%), *Pseudomonadaceae* (8.90%), *Oceanospirillaceae* (6.23%), and *Bacillaceae* (4.75%) (Figure S1D). These results indicate that coastal biofilm communities at Huiquan Bay are numerically dominated by *Gammaproteobacteria*, with *Vibrionaceae* representing the principal cultivable family.

Differences in bacterial recovery between media types are presented in Figure S2. A total of 204 isolates were obtained on MA, whereas 135 isolates were recovered on TCBS medium. The dominance of *Vibrio* spp. in the combined dataset reflects the selective nature of TCBS medium, which favors the growth of *Vibrio* and closely related taxa.

### 3.2. Seasonal Dynamics of the Cultivable Bacteria Isolated from MA

To assess community composition independent of selective *Vibrio* enrichment, MA-derived isolates were analyzed separately (Figure S3A–D). The MA community remained dominated by *Pseudomonadota* (79.41%), followed by *Bacillota* (14.71%), *Bacteroidota* (5.39%), and *Actinomycetota* (0.49%) (Figure S3A). At the class level, *Gammaproteobacteria* accounted for 69.12% of isolates (Figure S3B). Order-level analysis showed *Alteromonadales* (30.88%) and *Vibrionales* (20.10%) as dominant groups (Figure S3C), while family-level composition revealed *Vibrionaceae* (20.10%), Pseudoalteromonadaceae (16.18%), Moraxellaceae (14.71%), and *Alteromonadaceae* (10.29%) as major constituents (Figure S3D). These results demonstrate that MA cultivation captured a broader heterotrophic assemblage beyond vibrios.

**Figure 2:**
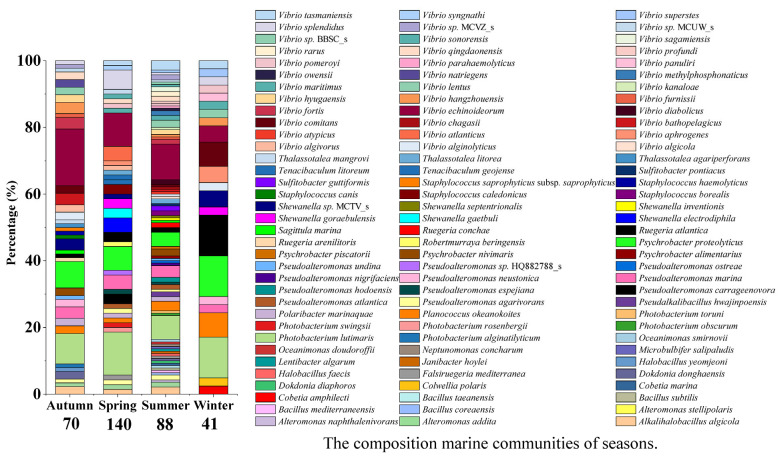
The seasonal compositions of marine bacterial communities. The bar chart displays the percentage composition of specific microbial species across four seasons: spring, summer, autumn, and winter.

Seasonal variation in isolate recovery was evident, with 70 isolates obtained in spring, 140 in summer, 88 in autumn, and 41 in winter, indicating that summer supported the highest cultivable bacterial abundance. Seasonal variation was also evident in the taxonomic composition of cultivable bacteria. *Pseudomonadota* dominated across all seasons, with the highest proportions in spring and summer, while *Bacteroidota* and *Bacillota* were more abundant in autumn and winter. *Gammaproteobacteria* were the predominant class throughout the year, particularly in spring, followed by summer, whereas *Bacilli* and *Flavobacteriia* contributed more noticeably in winter. At the family level, *Vibronaceae* showed marked dominance during spring and summer, coinciding with peaks in *Vibrio* abundance, while *Alteromonadaceae* was more common in autumn, and *Pseudomonadaceae* increased in winter. Genus-level analysis revealed that *Vibrio* was the dominant genus in spring and summer, whereas a variety of non-*Vibrio* genera were present across all seasons. In spring, these included *Pseudalteromonas* species (*P. undina*, *P. nigrifaciens*, *P. hodoensis*, *P. altaetica*), *Shewanella* species (*S. goraebulensis*, *Shewanella* sp. MCTV_s), *Photobacterium* species (*P. swingsii*, *P. lutimaris*), as well as *Tenacibaculum litorium*, *Sulfitobacter gutifrons*, *Ruegeria arenitioris*, *Psychrobacter pisciatori*, *Polaribacter marinauae*, *Oceanimonas doudoroffii*, *Lentibacter algarum*, *Halobacillus faecis*, *Dokdonia diaphoros*, *Cobetia amphilecti*, *Bacillus mediterraneans*, *Alteromonas naphthalenivorans*, and *Thalassotalea magna*. Summer isolates showed a similar pattern, with non-*Vibrio* genera such as *Photobacterium*, *Alteromonas*, and *Pseudoalteromonas* remaining prevalent (Figure 2). In autumn and winter, the non-*Vibrio* community composition shifted. *Photobacterium* became relatively more abundant in autumn, whereas in winter, genera such as *Alteromonas*, *Pseudomonas*, and *Staphylococcus* contributed more prominently, alongside persistent low levels of *Vibrio*. Overall, these results demonstrate that family-level community structure is strongly shaped by season, with warmer periods favoring *Vibrionaceae* dominance, whereas cooler seasons support increased contributions from other heterotrophic bacterial families, including *Alteromonadaceae*, *Pseudomonadaceae*, *Staphylococcaceae*, and multiple genera within *Gammaproteobacteria*, *Bacteroidota*, and *Bacillota.*

### 3.3. Composition and Seasonal Dynamics of Vibrionaceae

**Figure 3:**
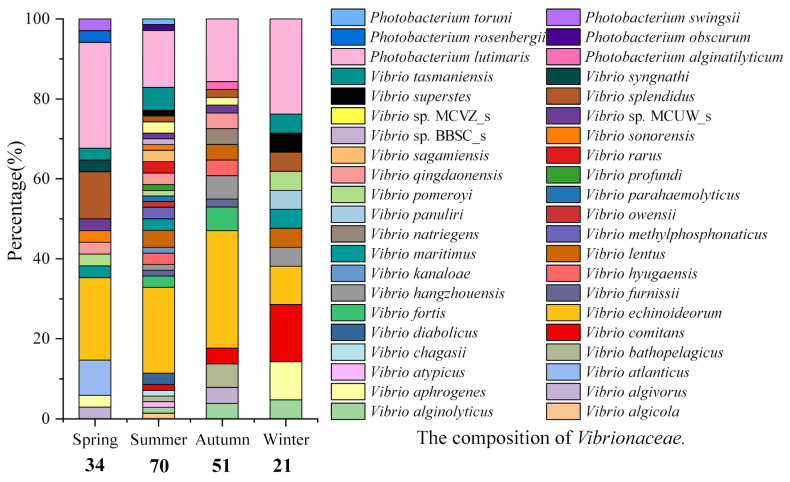
Seasonal composition of *Vibrionaceae* communities. The bar chart displays the percentage composition of specific microbial species across four seasons, i.e., spring, summer, autumn, and winter.

A total of 176 *Vibrionaceae* isolates were recovered across the four seasons, comprising 139 *Vibrio* isolates and 37 *Photobacterium* isolates (Figure 3). Among the *Vibrio* isolates, 105 were assigned to species, while the remaining were classified as *Vibrio* spp. due to insufficient 16S rRNA gene sequence similarity to reference type strains. *V. echinoideorum* was the most frequently recovered species and showed pronounced seasonal variability, being particularly abundant during spring and summer, with substantially fewer isolates detected in autumn and winter. *V. splendidus* showed higher representation during spring, whereas *V. comitans* was more frequently recovered from winter samples, indicating species-specific seasonal preferences. Members of the genus *Photobacterium* were detected throughout the year, with *Photobacterium lutimaris* representing the dominant non-*Vibrio* taxon and occurring in all seasons. Overall, these results demonstrate clear seasonal structuring within the community, with warmer periods favoring higher *Vibrio* abundance and species turnover. At the same time, cooler seasons were characterized by reduced *Vibrio* recovery and relatively increased contributions from non-*Vibrio* taxa.

### 3.4. Phylogenetic Tree Analysis

**Figure 4:**
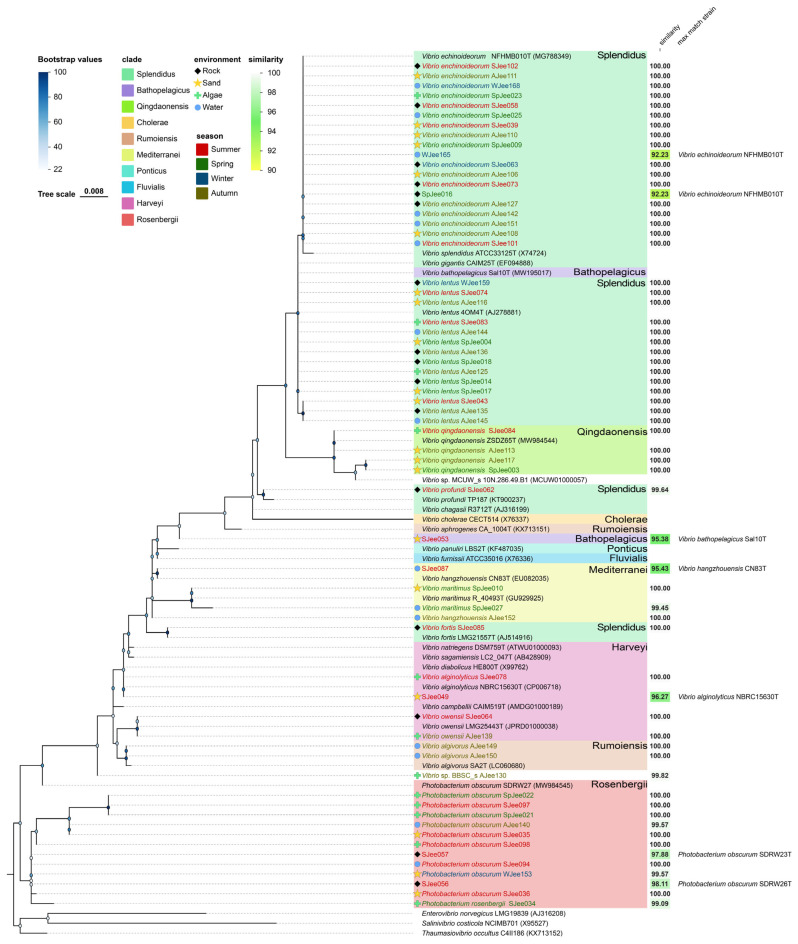
Phylogenetic analysis of *Vibrionaceae* isolates based on nearly the whole 16S rRNA gene sequences. *Enterovibrio norvegicus* LMG 19,839 (AJ316208), *Salinivibrio costicola* NCIMB 701 (X95527), and *Thaumasiovibrio occultus* C4II186 (KX713152) were used collectively as outgroup taxa, together with 27 validated *Vibrio* species type strains serving as reference sequences to provide a stable phylogenetic framework [31]. These basal lineages were used to polarize isolate clustering in the absence of a single designated outgroup. The resulting maximum-likelihood tree resolved isolates into several well-supported clades, including the *Splendidus*, *Harveyi*, *Mediterranei*, and *Qingdaonensis* clades, as well as a distinct *Photobacterium* lineage.

Phylogenetic analysis based on nearly full-length 16S rRNA gene sequences resolved the isolates into well-supported clades corresponding to the genera *Vibrio* and *Photobacterium*, with bootstrap values ≥ 70% at most major nodes (Figure 4). The maximum-likelihood tree was rooted using an appropriate outgroup, which formed a distinct basal branch. The majority of isolates clustered within the genus *Vibrio*, forming several monophyletic species-level and species-complex clades.

A prominent, closely related clade corresponding to the *V. echinoideorum* and *V. lentus* species complex was recovered and was among the most abundant lineages in the dataset. This clade comprised numerous isolates obtained from multiple substrates across all seasons, indicating broad ecological distribution. Several isolates showed reduced sequence similarity to the type strain *V. echinoideorum* NFHMB010, suggesting substantial genetic heterogeneity within this species complex.

The *Splendidus* clade represented the most abundant and phylogenetically diverse group among the *Vibrio* isolates. Most strains clustered tightly with reference strains of *V. echinoideorum* and *V. lentus*, exhibiting very high sequence similarity (typically approaching 100%). A small number of *V. echinoideorum* isolates displayed lower similarity (as low as 92.23%) but remained within the same clade. Isolates related to *V. profundi* were positioned within the broader *Splendidus* clade, consistent with their established phylogenetic relationships. In addition, isolates affiliated with the *Mediterranean* clade formed a distinct and well-supported lineage, clearly separated from other major *Vibrio* clades. Although less abundant than members of the *Splendidus* clade, *Mediterranean* clade isolates were consistently detected among the sampled coastal isolates, confirming their presence in this environment.

Several additional species-level clades were also resolved, including *V. qingdaonensis*, *V. hangzhouensis*, *V. maritimus*, *V. algivorus*, *V. owensii*, and *V. bathopelagicus*, with isolates clustering closely with their respective type strains. These consistent clustering patterns support the reliability of species-level assignments based on 16S rRNA gene sequences. Overall, the phylogenetic reconstruction demonstrates that the isolates are distributed among established *Vibrio* clades, with the *Splendidus* clade representing the most abundant and genetically diverse lineage, while the *Mediterranean* clade and other species-level groups form smaller, well-resolved lineages within the coastal biofilm community.

### 3.5. Dynamics of Biofilm Formation of Total Vibrio Isolates Across Seasons

**Figure 5:**
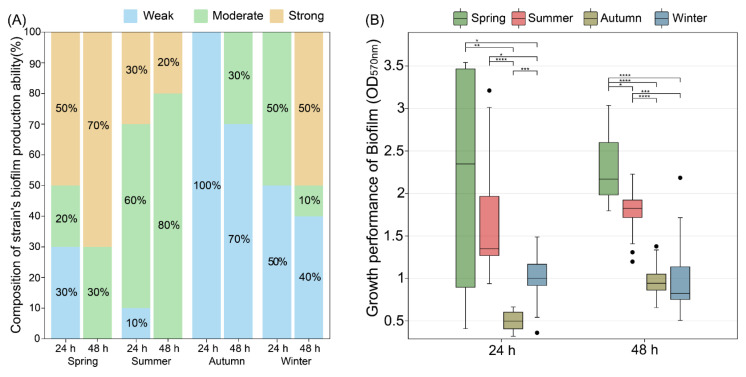
Seasonal variation in biofilm-forming capacity of *Vibrio* isolates. (**A**), Percentages of strains exhibiting weak, moderate, and strong biofilm formation at 24–48 h among seasons. Biofilm strength was classified based on OD_570nm_ readings, showing faster biofilm development in Spring and delayed formation in Autumn and Winter. (**B**), The differences in biofilm formation among seasons. Significant differences among seasons were assessed using Kruskal–Wallis tests followed by Dunn’s post-hoc comparisons with Bonferroni correction. Spring and summer isolates exhibited significantly higher biofilm formation than autumn isolates at both time points, while winter isolates showed intermediate levels. Asterisks indicate significance levels: ****, *p* < 0.001; ***, *p* < 0.01; **, *p* < 0.01; *, *p* < 0.05; ns = not significant.

Biofilm formation by representative *Vibrio* isolates was quantified after 24 h and 48 h incubation using crystal violet staining. To examine seasonal differences, we first analyzed the proportion of strong biofilm-forming isolates (Figure 5). The composition of strains based on biofilm production ability varied markedly across seasons (Table S1 and Figure 5A). Spring isolates exhibited rapid biofilm development, with half of the strains already forming strong biofilms at 24 h, increasing to 70% after 48 h. Autumn isolates displayed delayed biofilm maturation, with only 30% classified as strong biofilm formers, increasing to 70% at 48 h. Summer isolates exhibited moderate, relatively stable biofilm formation across both time points. Winter isolates initially had a high proportion of strong biofilm producers at 24 h (50%), followed by a marked decline to 10% at 48 h, indicating reduced biofilm stability over time.

Statistical analysis using Kruskal–Wallis tests followed by Dunn’s post-hoc comparisons with Bonferroni correction confirmed that season significantly affected biofilm formation. (24 h: χ^2^ = 16.131, df = 3, *p* = 0.001; 48 h: χ^2^ = 30.394, df = 3, *p* < 0.001) (Table S2 and Figure 5B). Pairwise comparisons indicated that isolates from spring and summer exhibited significantly greater biofilm-forming capacity than those from autumn at both time points. Winter isolates showed intermediate levels and did not differ significantly from other seasons. No significant differences were detected between spring and summer or between autumn and winter.

### 3.6. Substrate Patterns of Vibrio Biofilm Formation

**Figure 6:**
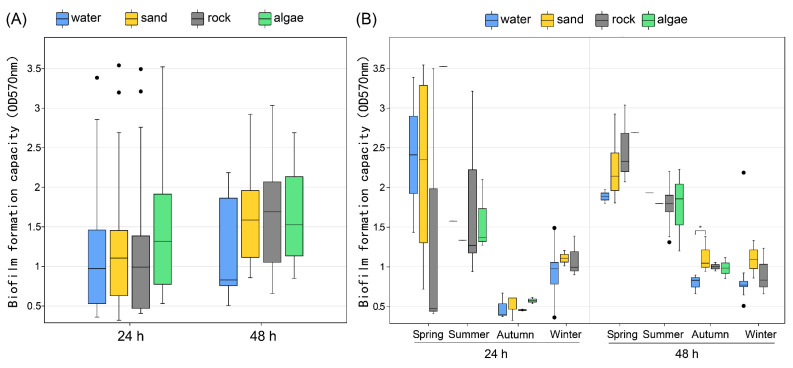
Substrate-associated variation in biofilm formation of *Vibrio* isolates. (**A**), Percentages of strains exhibiting weak, moderate, and strong biofilm formation among substrates. No significant differences were detected among substrate types at either incubation time (Kruskal–Wallis test, *p* > 0.05). (**B**), The differences in biofilm formation among substrates. A few differences were detected among substrate types at 48 h. Asterisks indicate significance levels: *, *p* < 0.05.

Substrate type was evaluated, but no significantly affect biofilm formation at either incubation time (24 h: χ^2^ = 0.780, df = 2, *p* = 0.677; 48 h: χ^2^ = 0.032, df = 2, *p* = 0.984) (Table S3 and Figure 6A,B). Because the overall tests were not significant, post-hoc pairwise comparisons were not performed. These results indicate that seasonal origin, rather than substrate type, was the primary factor influencing biofilm formation under the experimental conditions.

Although substrate type did not show statistical significance, a descriptive trend in mean biofilm formation was observed, generally following the pattern rock > algae > water > sand. Sand-associated isolates consistently exhibited the lowest biofilm formation at both time points, suggesting that the unstable and nutrient-poor nature of sandy substrates may limit bacterial attachment and biofilm maturation. Rock-associated isolates showed relatively stable and moderate biofilm formation across seasons. Algal substrates displayed strong seasonal dependence: spring and summer isolates formed robust biofilms (with several strains showing OD_570nm_ > 3.0), whereas winter isolates exhibited minimal development. Water-associated isolates showed high variability, with some spring strains forming strong biofilms and winter strains showing weak formation.

Strain-level variation further contributed to these patterns. Representative strong biofilm-forming strains included spring isolates such as *V. splendidus* and *V. echinoideorum*, whereas no strong biofilm formers were detected among autumn isolates. Seasonal differences were pronounced. Spring isolates exhibited the highest overall biofilm-forming capacity, with a large proportion classified as strong at both 24 h and 48 h. Summer isolates displayed a mixed pattern, with some strong biofilm formers but most strains remaining moderate. Autumn isolates were uniformly weak at 24 h, and although some reached moderate levels at 48 h, no strong biofilm formation was observed. Winter isolates generally showed weak biofilm formation, except for one strain that demonstrated strong biofilm formation at 48 h, indicating limited and strain-specific biofilm potential during winter.

### 3.7. Biofilm Formation Capacity of Vibrio Isolates

**Figure 7:**
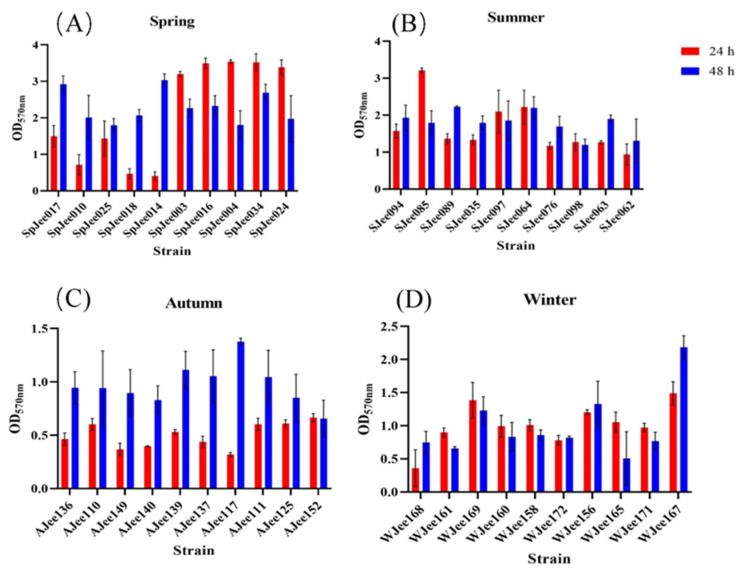
Seasonal variation in biofilm formation by separated *Vibrio* species. Biofilm formation was quantified by crystal violet staining and measured as OD_570nm_ after 24 and 48 h of incubation. The biofilm formation of *Vibrio* strains isolated from Qingdao in spring (**A**), summer (**B**), autumn (**C**), and winter (**D**).

Based on preliminary screening, 40 representative strains (10 per season) were selected for quantitative biofilm analysis. These strains, isolated from four substrates (rock, sand, algae, and water) across all seasons, were assessed for biofilm formation at 24 h and 48 h using crystal violet staining (Table S4 and Figure 7). Considerable variability in biofilm-forming capacity was observed among species and individual strains. Spring isolates (Figure 7A) exhibited the strongest overall biofilm formation among all seasons. Several *V. echinoideorum* strains (SpJee017, SpJee025, SpJee014) demonstrated clear increases in biofilm formation from 24 to 48 h, indicating progressive maturation. *V. sonorensis* (SpJee010) and *V. atlanticus* (SpJee018) shifted from moderate biofilm formation at 24 h to stronger production at 48 h. Two *V. splendidus* strains showed high biofilm formation at 24 h; one maintained elevated levels at 48 h, whereas the other displayed a reduction. Additional spring isolates, including *V. aphrogenes*, *Vibrio.* sp. MCUW_s and *Photobacterium rosenbergii*, also exhibited strong biofilm formation, highlighting pronounced inter-strain variability within this season.

Summer isolates (Figure 7B) were predominantly moderate biofilm producers. *Photobacterium lutimaris* strains displayed relatively stable biofilm production between 24 and 48 h. *V. fortis* isolates showed variable temporal patterns, with some increasing and others decreasing over time. *V. parahaemolyticus* maintained consistently high biofilm formation at both incubation times. Other species, including *V. alginolyticus*, *V. echinoideorum*, and *V. profundi*, generally exhibited moderate biofilm production with limited temporal variation.

Autumn isolates (Figure 7C) were characterized mainly by weak to moderate biofilm formation. Species such as *V. lentus*, *V. echinoideorum*, *V. algivorus*, and *Photobacterium lutimaris* showed only minor increases between 24 and 48 h. *V. natriegens* and *V. owensii* increased from weak to moderate biofilm formation at 48 h. Notably, *Vibrio* sp. MCUW_s (AJee117) demonstrated a marked increase in biofilm formation over time, whereas most other autumn isolates remained weak biofilm producers.

Winter isolates (Figure 7D) exhibited comparatively low to moderate biofilm formation. Most strains showed limited temporal variation between 24 and 48 h. A small number of isolates, including *V. comitans* (WJee167), displayed increased biofilm production at 48 h, whereas others, such as *V. splendidus*, showed reduced biomass over time. Strong biofilm formation was observed in only a few winter strains. Overall, biofilm formation patterns varied across seasons, with higher biofilm formation generally observed in spring and summer isolates compared with autumn and winter isolates.

## 4. Discussion

This study provides a cultivation-based characterization of *Vibrionaceae* inhabiting coastal marine biofilms across four seasons, integrating species diversity, phylogenetic structure, and functional biofilm traits. By combining selective and non-selective media with phylogenetic identification and quantitative biofilm assays, we demonstrate that seasonal variability and strain-level heterogeneity jointly structure *Vibrio* community composition and ecological function in a dynamic coastal environment.

### 4.1. Species Diversity, Phylogenetic Structure, and Ecological Strategies of Vibrionaceace in Biofilm Formation

The *Vibrionaceae* community associated with coastal biofilms exhibited clear seasonal patterns at the species level, together with substantial variation in biofilm-forming capacity among isolates [5,32]. In the present study, certain taxa, such as *V. echinoideorum*, were more frequently detected during spring and summer, whereas members of the *V. splendidus* clade persisted throughout all four seasons. For example, *V. splendidus* was recovered year-round, while *V. lentus* was absent in spring but present during summer, autumn, and winter. These findings indicate that *Vibrionaceae* populations within coastal biofilms include both seasonally responsive taxa and persistent lineages [32,33]. Seasonal shifts in *Vibrio* populations are widely recognized to be influenced by environmental factors, particularly temperature [16,22]. Previous studies have shown that elevated seawater temperatures promote the proliferation of certain *Vibrio* species, whereas other lineages exhibit broader thermal tolerance and persist across seasons. The patterns observed in this study are broadly consistent with these findings; however, because environmental parameters were not directly measured, the role of temperature is inferred based on previous studies rather than established through direct analysis.

Phylogenetic analysis based on nearly full-length 16S rRNA gene sequences further resolved [34] that the isolates are divided into several major clades, including the *Splendidus*, *Harveyi*, *Mediterranean*, and *Orientalis* lineages, together with a distinct *Photobacterium* cluster [35,36] (Figure 4). The *Splendidus* clade represented the most diverse and seasonally persistent lineage, particularly during spring and winter. The coexistence of multiple phylogenetic lineages within biofilms further indicates that phylogenetic diversity contributes to functional redundancy and ecological stability in surface-associated communities [37].

Similar strain-level variability in biofilm formation has been reported in other *Vibrio* species, highlighting the role of microdiversity in shaping functional traits [38]. Some strains consistently produced high biofilm formation, whereas others remained weak biofilm formers regardless of seasonal origin [39]. This intraspecific variability demonstrates that biofilm phenotype cannot be inferred solely from species identity [40,41]. Instead, strain-specific genetic composition and regulatory mechanisms likely play critical roles in determining surface colonization and biofilm development [5]. The elevated biofilm formation observed during warmer seasons may therefore reflect a combination of environmental stimulation and seasonal enrichment of strains with intrinsically strong biofilm-forming potential [42]. Conversely, the greater representation of weak biofilm formers in cooler seasons suggests that seasonal environmental conditions may influence not only species composition but also functional structure at the subpopulation level [39,43].

Overall, the integration of species distribution patterns, phylogenetic structure, and strain-level functional variability reveals a highly organized *Vibrionaceae* assemblage within coastal marine biofilms [38,44]. Seasonal environmental drivers, together with clade-specific traits and intraspecific heterogeneity, appear to collectively shape community dynamics and contribute to the stability and adaptability of these biofilm-associated populations.

### 4.2. Seasonal Regulation of Vibrio Biofilm Formation in Coastal Environments

Biofilm formation by representative *Vibrio* isolates was quantified after 24 h and 48 h of incubation using crystal violet staining (Figure 5A,B), and seasonal differences were evaluated using Kruskal–Wallis tests followed by Dunn’s post-hoc comparisons with Bonferroni correction At both 24 h and 48 h, biofilm formation differed significantly among seasons (24 h: χ^2^ = 16.131, df = 3, *p* = 0.001; 48 h: χ^2^ = 30.394, df = 3, *p* < 0.001). Isolates obtained during spring and summer exhibited significantly higher biofilm-forming capacity than those recovered in autumn, while winter isolates showed intermediate levels [45] No significant differences were detected between spring and summer, or between autumn and winter [46]. These results indicate that warmer-season conditions favor biofilm development among coastal *Vibrio* populations.

Temperature likely represents a primary environmental driver underlying this seasonal regulation [47]. *Vibrio* species are well recognized for their temperature-dependent growth dynamics, and numerous studies have demonstrated that elevated temperatures stimulate metabolic activity, exopolysaccharide production, and quorum-sensing pathways associated with biofilm formation [48,49]. Increased membrane fluidity and enzymatic activity at higher temperatures may accelerate surface attachment and matrix synthesis, facilitating more rapid biofilm maturation [50]. The stronger biofilm formation observed among spring and summer isolates is therefore consistent with temperature-mediated regulation of biofilm-associated gene expression [51]. In addition to short-term physiological responses, seasonal warming may selectively enrich strains with inherently greater biofilm-forming capacity, reinforcing observed community-level differences [52].

Seasonal fluctuations in nutrient availability may also play a significant role in regulating biofilm development and structure [39,53]. Warmer months are often associated with elevated primary productivity and phytoplankton blooms, increasing dissolved organic carbon and polysaccharide availability in coastal waters [54,55]. Organic substrates derived from phytoplankton exudates can serve as carbon sources and chemical signals that promote bacterial attachment and extracellular polymeric substance production [56]. Enhanced organic inputs during productive seasons may thus create favorable microhabitats for surface colonization and biofilm persistence [5,57].

Hydrodynamic conditions represent another potential regulatory factor [58]. Greater water column stratification during warmer periods may stabilize surface-associated communities, whereas stronger mixing and turbulence during colder seasons can disrupt early biofilm formation and favor planktonic dispersal [5,59]. The interaction between physical stability and physiological temperature responses likely contributes to the seasonal biofilm patterns observed in this study [39,53]. Importantly, substantial strain-level heterogeneity was observed within each season. Although *V. echinoideorum* was detected across all seasons and substrates, biofilm development varied markedly among isolates. Even within the same species, certain strains maintained strong biofilm formation between 24 h and 48 h, whereas others showed reduced biomass over time. This intraspecific variability underscores the strain-dependent nature of biofilm formation and suggests that seasonal environmental filtering operates not only at the species level but also at the genotype level [60,61]. Such fine-scale functional differentiation may enhance ecological resilience by maintaining diverse colonization strategies within coastal *Vibrio* populations [38,62].

Overall, seasonal environmental variability appears to regulate *Vibrio* biofilm formation through integrated effects of temperature, nutrient dynamics, hydrodynamic stability, and strain-specific physiological capacity, consistent with broader models of environmental filtering in marine microbial communities [5,6,63].

### 4.3. Influence of Substrate Characteristics on Vibrio Biofilm Development

In contrast to seasonal effects, substrate type did not significantly affect biofilm formation at either incubation time (24 h: χ^2^ = 0.780, df = 2, *p* = 0.677; 48 h: χ^2^ = 0.032, df = 2, *p* = 0.984). Because the overall statistical tests were not significant, post-hoc pairwise comparisons were not performed. These results indicate that, under standardized laboratory conditions, seasonal origin exerted a stronger influence on biofilm formation than substrate identity.

Despite the absence of statistically significant differences in the experimental system, substrate characteristics are widely recognized as important regulators of biofilm development under natural conditions [41,64]. Previous studies have reported that isolates associated with algal and rocky substrates often exhibit stronger and more stable biofilm formation than those derived from sand or water-column habitats [56,65]. Benthic substrates provide relatively stable attachment surfaces and may release organic compounds that promote bacterial adhesion and extracellular polymeric substance production [56,66]. In particular, macroalgal surfaces release polysaccharides and dissolved organic matter that function as chemoattractants and carbon sources, facilitating microbial colonization [5,56]. Surface roughness and mineral composition of rocky substrates can further enhance cell retention and biofilm maturation [65,67]. In contrast, sand particles often have smoother surfaces and greater physical disturbance, which may limit the establishment of stable biofilms [57].

Although substrate type did not significantly influence overall biofilm formation in the experimental system, previous studies have demonstrated that substrate origin can strongly regulate biofilm performance under natural environmental conditions. In natural coastal ecosystems, substrate-specific responses are often modulated by interactions among physicochemical gradients, hydrodynamics, and biological activity [68].

Benthic habitats have been proposed to function as environmental reservoirs for *Vibrio* populations, supporting persistence during unfavorable periods, whereas water-column-associated populations may represent dispersal phases within the *Vibrio* life cycle [69]. These findings suggest that *Vibrio* biofilm formation is structured by a hierarchy of ecological drivers [38].

### 4.4. Limitations and Future Directions

Despite these findings, it is important to recognize that this study is based on culture-dependent methods, which inherently capture only a small fraction of environmental microbial diversity. As a result, the *Vibrionaceae* diversity described here likely underrepresents the true community composition in coastal biofilms. Although cultivation allows detailed functional analyses, including biofilm phenotyping, it does not fully resolve the taxonomic and functional complexity of natural microbial assemblages. Future studies combining culture-based approaches with culture-independent techniques, such as amplicon sequencing and metagenomics, will be essential to achieve a more comprehensive and mechanistic understanding of *Vibrionaceae* ecology in coastal systems.

In addition, although the methodological approaches used in this study are well established, their application across multiple substrates and seasons provides comparative insights into the distribution and biofilm-forming capacity of cultivable *Vibrionaceae*. Nevertheless, the use of culture-based and 16S rRNA gene approaches limits resolution at the functional and genomic levels. Future studies integrating advanced genomic and transcriptomic approaches would enable a more comprehensive understanding of the ecological roles and adaptive mechanisms of *Vibrionaceae* in marine biofilm systems.

A limitation of this study is the absence of concurrent environmental measurements (e.g., temperature, salinity, and light intensity), which restricts direct evaluation of physicochemical drivers underlying seasonal variation. Therefore, interpretations of environmental influence are based on previously reported studies rather than site-specific data. Future work integrating environmental monitoring with microbial and functional analyses will be essential to establish robust links between environmental variability and biofilm dynamics.

## 5. Conclusions

In this study, we investigated the seasonal distribution, phylogenetic diversity, and biofilm-forming capacity of cultivable *Vibrionaceae* across multiple coastal substrates. The results demonstrated clear seasonal variation in biofilm formation, with isolates from warmer seasons generally exhibiting stronger biofilm-forming capacity. Substrate type also influenced biofilm development, with stable surfaces such as algae and rocks supporting more consistent biofilm formation than sand and glass plate-associated biofilms. Phylogenetic analysis indicated that biofilm-forming capacity was not restricted to specific lineages but was distributed across multiple clades, suggesting substantial strain-level variability. Collectively, these findings indicate that *Vibrionaceae* biofilm formation is influenced by multiple interacting ecological mechanisms, including seasonal environmental variation, substrate heterogeneity, and strain-level differences in biofilm-forming capacity. These findings provide insights into the distribution patterns and functional characteristics of cultivable *Vibrionaceae* in coastal biofilm systems. However, because this study is based on culture-dependent methods and lacks direct environmental measurements, the ecological interpretations remain limited. Future studies integrating culture-independent approaches (e.g., amplicon sequencing, metagenomics) with concurrent environmental monitoring will be essential for a more comprehensive understanding of *Vibrionaceae* ecology in marine environments.

## Data Availability

The original contributions presented in this study are included in the article/Supplementary Material. Further inquiries can be directed to the corresponding authors.

## Supplementary Materials

The following supporting information can be downloaded at: https://www.mdpi.com/article/10.3390/microorganisms14061259/s1, Table S1: Kruskal–Wallis test comparisons of biofilm formation across seasons at 24 h incubation (including Dunn’s post-hoc with Bonferroni correction); Table S2: Kruskal–Wallis test comparisons of biofilm formation across seasons at 48 h incubation (including Dunn’s post-hoc with Bonferroni correction); Table S3: Kruskal–Wallis test results for biofilm formation across substrates at 24 h and 48 h incubation; Table S4. Identification and biofilm-forming capacity (24 h and 48 h) of representative Vibrio and Photobacterium isolates across seasons; Figure S1: Taxonomic composition of cultivable bacterial families at phylum, class, order, and family levels (stacked bar charts); Figure S2: Differences in bacterial recovery between MA and TCBS media; Figure S3: Seasonal dynamics of MA-derived bacterial communities (relative abundance bar charts).
