# Supplementary material for: Preliminary Insights into the Seasonal Variation, Phylogenetic Diversity, and Biofilm-Forming Capacity of Cultivable Vibrionaceae in Coastal Biofilms of Qingdao, China
Source: Microorganisms. 2026 Jun 3;14(6):1259. doi: 10.3390/microorganisms14061259 (PMC13304392; doi:10.3390/microorganisms14061259)
Supplement: Supplementary file 1 [file microorganisms-14-01259-s001.zip › microorganisms-4233678-supplementary.pdf]

Supplementary materials for:

Table S1. Kruskal–Wallis test and Dunn’s post-hoc comparisons of biofilm formation across seasons at 24 h Incubation

Comparison	Z value	Raw *p*-value	Adjusted *p*-value	Significance
Autumn–Winter	-1.722	0.085	0.511	ns
Autumn–Spring	-3.635	<0.001	0.002	**
Autumn–Summer	-3.826	<0.001	0.001	**
Winter–Spring	1.913	0.056	0.335	ns
Winter–Summer	2.104	0.035	0.212	ns
Spring–Summer	-0.191	0.848	1	Ns

Sample size: n = 10 per season (total n = 40). Overall test: Kruskal–Wallis test: $\chi^2 = 16.131$, df=3, $p = 0.001$. Values represent Dunn’s test Z-statistics with a Bonferroni correction for multiple comparisons. Significance levels: *** $p < 0.001$; ** $p < 0.01$; * $p < 0.05$; ns = not significant.

Table S2. Kruskal–Wallis test and Dunn’s post-hoc comparisons of biofilm formation across seasons at 48 h incubation

Comparison	Z value	Raw *p*-value	Adjusted *p*-value	Significance
Autumn–Winter	-0.784	0.433	1	ns
Autumn–Spring	-4.036	<0.001	<0.001	***
Autumn–Summer	-2.64	0.008	0.05	*
Winter–Spring	4.821	<0.001	<0.001	***
Winter–Summer	3.424	0.001	0.004	**
Spring–Summer	1.396	0.163	0.976	Ns

Sample size: n = 10 per season (total n = 40). Overall test: Kruskal–Wallis test: $\chi^2 = 30.394$, df=3, $p = 0.001$. Values represent Dunn’s test Z-statistics with a Bonferroni correction for multiple comparisons. Significance levels: *** $p < 0.001$; ** $p < 0.01$; * $p < 0.05$; ns = not significant.

Table S3. Kruskal–Wallis test results for biofilm formation across substrates at 24 h and 48 h incubation

Time Point	n	χ^2	df	*p*-value	Post-hoc comparison
24 hrs	29	0.78	2	0.677	Not performed
48 hrs	29	0.032	2	0.984	Not performed

Post-hoc Dunn's comparisons were not conducted because the overall Kruskal–Wallis tests were not significant ($p > 0.05$).

Table S1. Identification and biofilm-forming capacity (24 h and 48 h) of representative *Vibrio* and *Photobacterium* isolates across seasons

Strain	Season	Genus	Similarity (%)	Medium	Treatment time						Substrate
					Biofilm_24h			Biofilm_48h			
SpJee017	Spring	*Vibrio echinoideorum*	98.9	MA	1.787	1.196	0.658	2.79	2.794	3.18	Sand
SpJee010	Spring	*Vibrio sonorensis*	98.9	MA	0.461	1.006	0.682	1.337	2.508	2.19	Sand
Sp[1]Jee025	Spring	*Vibrio echinoideorum*	97.67	TCBS	0.931	1.892	1.476	1.71	2.01	1.67	Water
SpJee018	Spring	*Vibrio atlanticus*	98.65	TCBS	0.346	0.617	0.446	1.925	2.239	2.04	Rock
SpJee014	Spring	*Vibrio echinoideorum*	97.5	TCBS	0.323	0.358	0.543	3.053	3.191	2.86	Rock
SpJee003	Spring	*Vibrio*_sp. MCUW_s	99.17	TCBS	3.151	3.158	3.286	2.006	2.301	2.5	Sand
SpJee016	Spring	*Vibrio splendidus*	90.96	TCBS	3.577	3.332	3.572	2.29	2.068	2.63	Rock
SpJee004	Spring	*Vibrio splendidus*	97.56	TCBS	3.585	3.549	3.488	1.492	1.67	2.25	Sand
SpJee034	Spring	*Photobacterium rosenbergii*	96.6	TCBS	3.251	3.64	3.674	2.749	2.434	2.89	Algae
SpJee024	Spring	*Vibrio aphrogenes*	99.43	TCBS	3.616	3.24	3.297	1.667	1.543	2.71	Water
SJee094	Summer	*Photobacterium lutimarus*	97.46	MA	1.755	1.58	1.387	1.951	1.574	2.27	Water
SJee85	Summer	*Vibrio fortis*	99.05	TCBS	3.156	3.191	3.2853	1.7	2.158	1.52	Rock
SJee089	Summer	*Vibrio splendidus*	96.56	TCBS	1.23	1.497	1.3711	2.212	2.26	2.21	Algae
SJee035	Summer	*Photobacterium lutimarus*	99	MA	1.448	1.186	1.3646	1.635	1.752	2	Sand
SJee097	Summer	*Vibrio methylphosphonaticus*	97.77	TCBS	1.489	2.151	2.6505	1.328	2.387	1.85	Algae
SJee064	Summer	*Vibrio parahemolyticus*	100	TCBS	2.091	2.727	1.8455	1.909	2.507	2.18	Rock
SJee076	Summer	*Vibrio alginolyticus*	97.43	TCBS	1.193	1.074	1.2517	1.957	1.399	1.72	Rock
SJee098	Summer	*Photobacterium lutimarus*	97.29	MA	1.38	1.425	1.0087	1.029	1.237	1.33	Algae

[1]

SJee063	Summer	*Vibrio echinoideorum*	97.99	TCBS	1.218	1.295	1.2926	1.782	1.951	1.97	Rock
SJee062	Summer	*Vibrio profoundii*	99.71	MA	0.646	1.216	0.955	1.896	1.31	0.72	Rock
AJee136	Autumn	*Vibrio lentus*	100	TCBS	0.514	0.474	0.399	0.807	0.92	1.11	Rock
AJee110	Autumn	*Vibrio echinoideorum*	99.06	TCBS	0.653	0.608	0.546	1.003	1.255	0.57	Sand
AJee149	Autumn	*Vibrio algivorus*	97.44	MA	0.419	0.379	0.305	0.642	1.002	1.04	Water
AJee140	Autumn	*Photobacterium lutimaris*	99.14	TCBS	0.398	0.4	0.392	0.981	0.73	0.78	Water
AJee139	Autumn	*Vibrio hyugaensis*	99.71	TCBS	0.506	0.536	0.55	1.014	1.314	1.01	Algae
AJee137	Autumn	*Vibrio natriegens*	97.43	TCBS	0.483	0.38	0.449	1.321	1.006	0.83	Rock
AJee117	Autumn	*Vibrio* sp. MCUW_s	99.71	TCBS	0.298	0.327	0.333	1.382	1.408	1.34	Sand
AJee111	Autumn	*Vibrio echinoideorum*	98.37	TCBS	0.57	0.669	0.567	0.894	1.335	0.9	Sand
AJee125	Autumn	*Vibrio echinoideorum*	99.13	MA	0.577	0.602	0.648	0.615	1.054	0.88	Algae
AJee152	Autumn	*Vibrio hangzhounsis*	98.99	TCBS	0.638	0.646	0.708	0.848	0.602	0.52	Water
WJee168	Winter	*Vibrio tasmaniensis*	99.15	MA	0.473	0.044	0.563	0.579	0.75	0.91	Water
WJee161	Winter	*Vibrio aphrogenes*	99.43	TCBS	0.84	0.972	0.882	0.679	0.627	0.67	Rock
WJee169	Witer	*Vibrio panuliri*	98.14	TCBS	1.109	1.402	1.645	1.123	1.101	1.47	Rock
WJee160	Winter	*Vibrio aphrogenes*	99.71	TCBS	0.833	1.162	0.982	0.943	0.578	0.97	Rock
WJee158	Winter	*Vibrio hangzhouensis*	99.57	MA	0.937	0.992	1.097	0.841	0.944	0.79	Sand
WJee172	Winter	*Vibrio alginolyticus*	99	MA	0.863	0.754	0.724	0.824	0.839	0.79	Water
WJee156	Winter	*Vibrio pomeroyi*	100	TCBS	1.168	1.202	1.242	0.934	1.566	1.48	Sand
WJee165	Winter	*Vibrio splendidus*	90.96	MA	1.187	1.086	0.887	0.041	0.768	0.71	Water
WJee171	Winter	*Vibrio comitans*	100	TCBS	1.046	0.936	0.935	0.915	0.652	0.74	Water
WJee167	Winter	*Vibrio comitans*	99.71	TCBS	1.429	1.351	1.685	2.387	2.09	2.08	Water

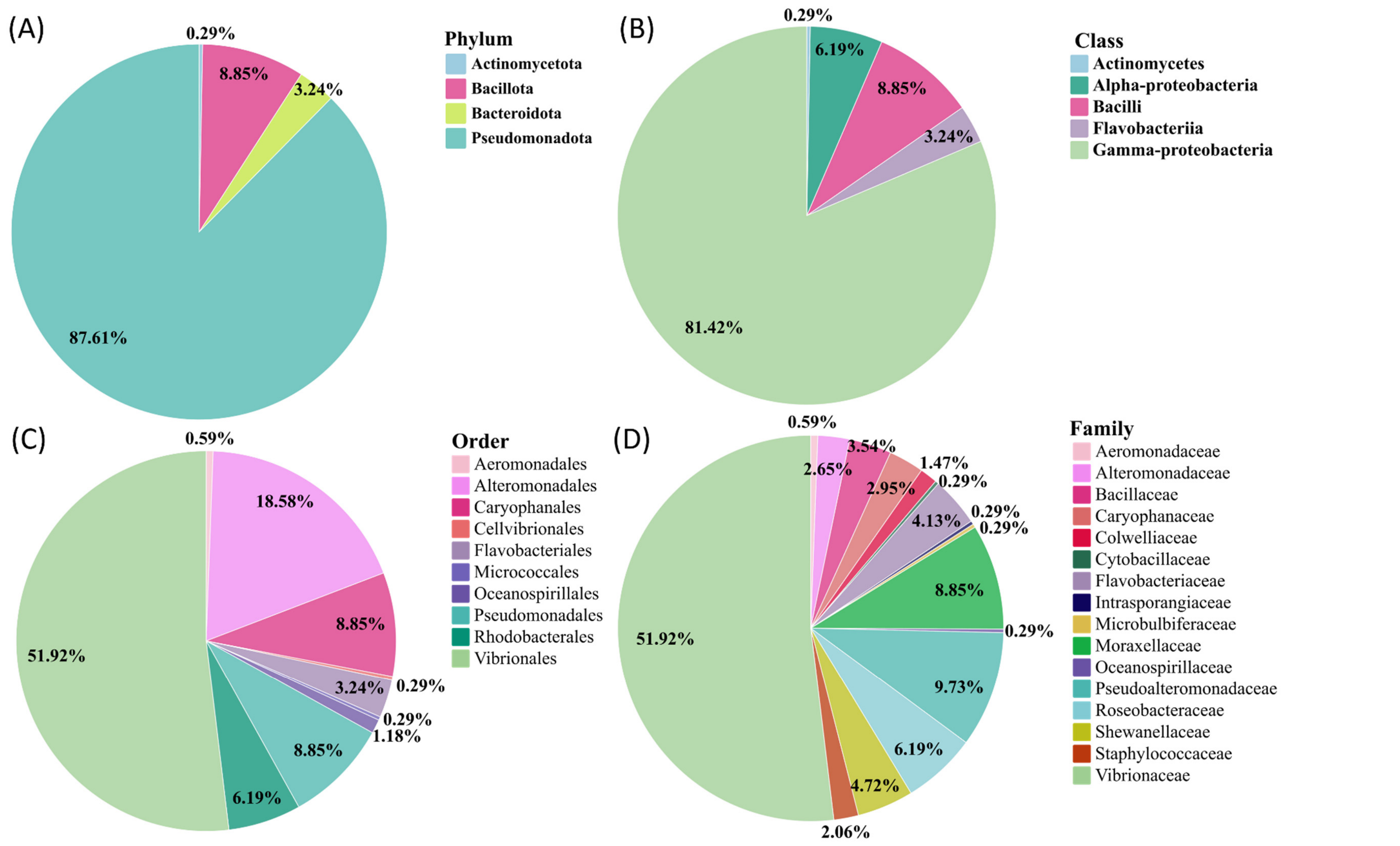

Figure S1 Taxonomic composition of total isolates. **(A)**, Phylum-level composition of the cultivable strains isolated from MA and TCBS; **(B)**, Class-level; **(C)**, Order-level; **(D)**, Family-level. Relative abundances are expressed as percentages of the total isolates.

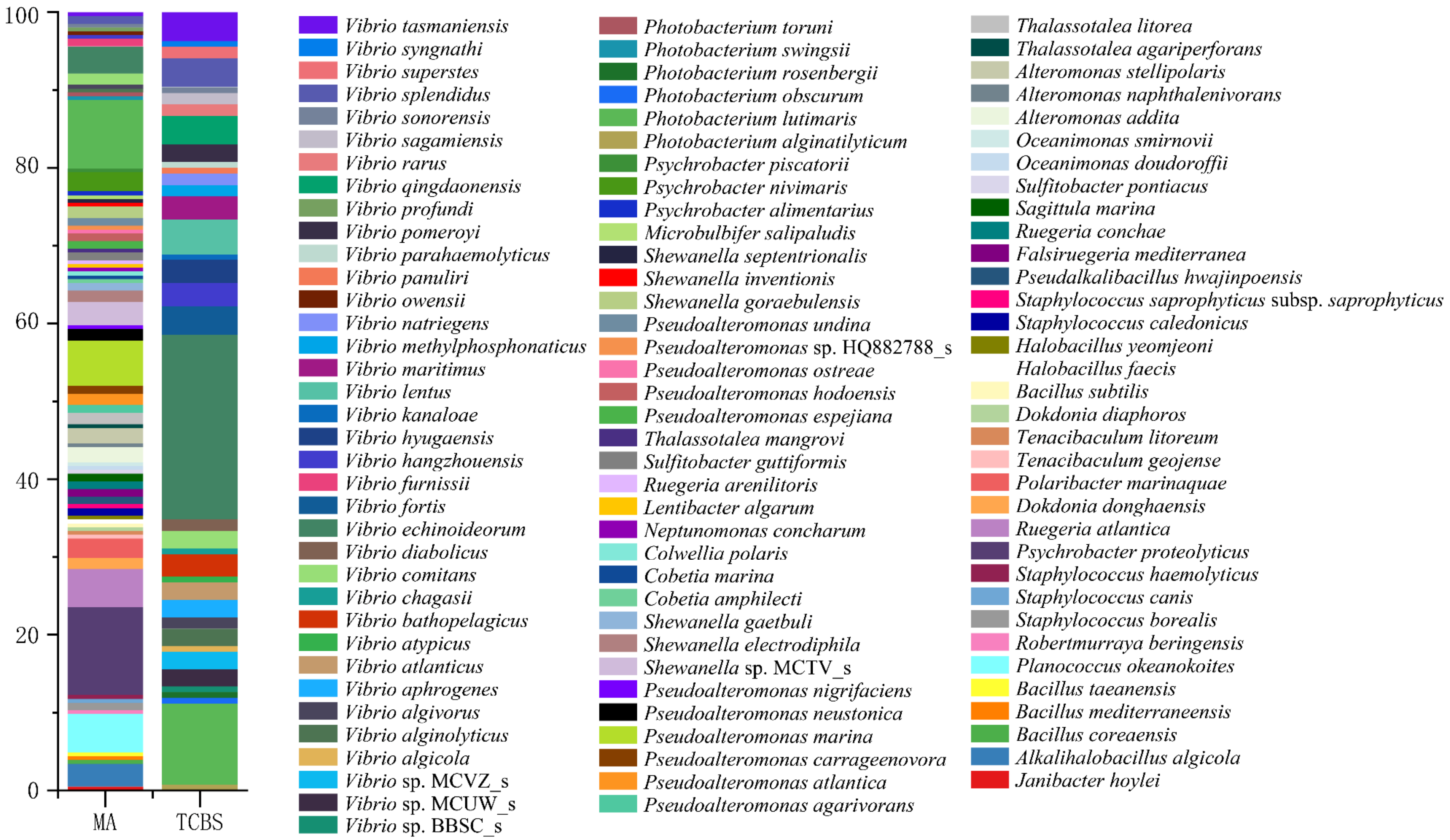

Figure S2**.** Selectivity of growth media on the composition of cultured marine bacteria. This stacked bar chart compares the relative abundance of bacterial

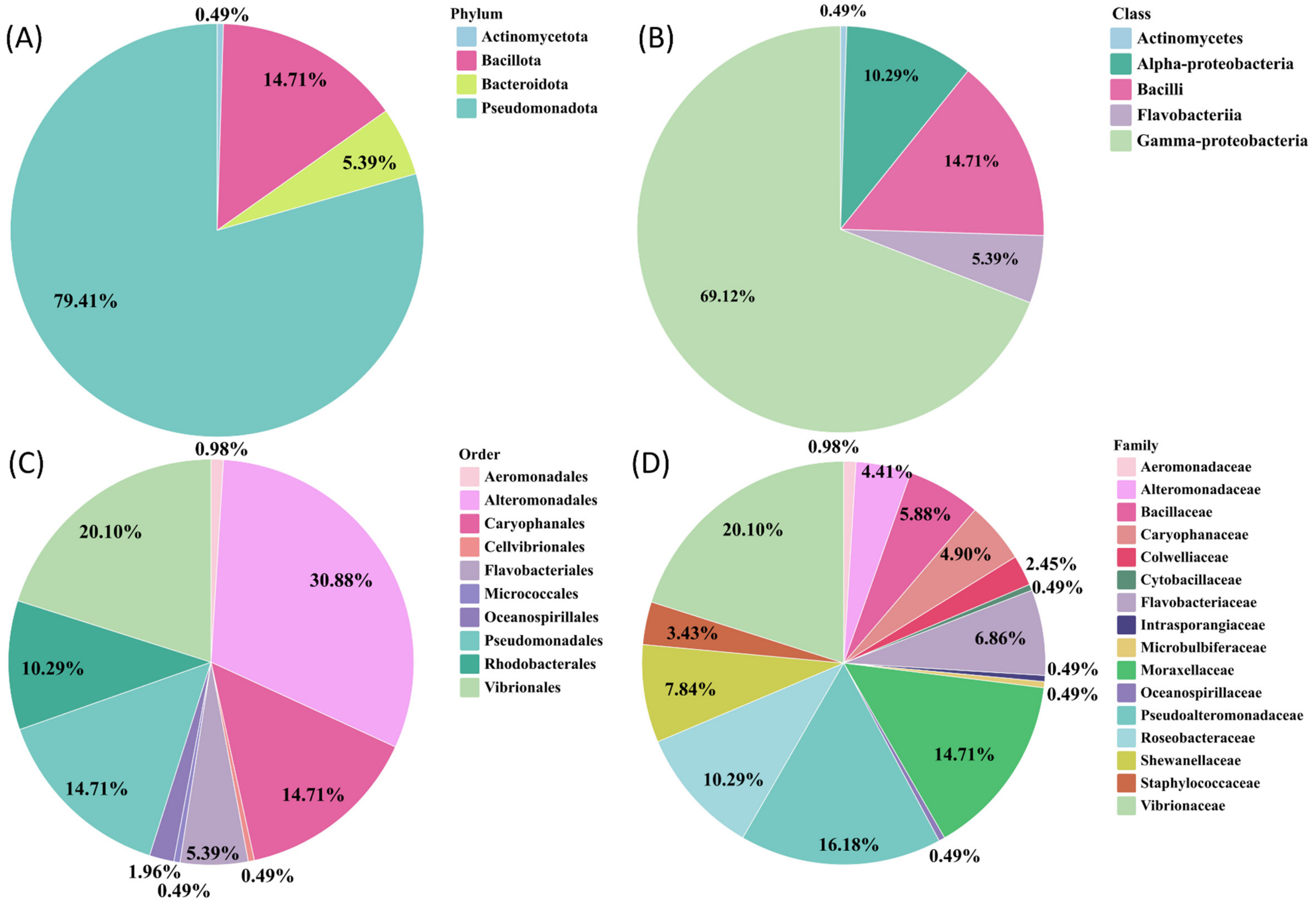

Figure S3**.** Taxonomic composition of cultivable bacteria recovered from MA. Phylum-level composition of the cultivable bacteria **(A)**; **(B)**, Class-level; **(C)**, Order-level; **(D)**, Family-level. Relative abundances are expressed as percentages of the total isolates.
